# Computational identification of biologically functional non-hairpin GC-helices in human Argonaute mRNA

**DOI:** 10.1186/1471-2105-14-122

**Published:** 2013-04-10

**Authors:** Simon Dornseifer, Georg Sczakiel

**Affiliations:** 1 Institut für Molekulare Medizin, Center for Structural and Cell Biology in Medicine (CSCM), Universität zu Lübeck, Ratzeburger Allee 160, Lübeck, D-23538, Germany; 2 Graduate School for Computing in Medicine and Life Sciences (GS-CMLS), Universität zu Lübeck, Ratzeburger Allee 160, Lübeck, D-23538, Germany

**Keywords:** GC-rich helix, Non-hairpin, RNA structure, Gene expression, Human argonaute

## Abstract

**Background:**

Perfectly formed duplex elements in RNA occur within folding units, often as a part of hairpin motifs which can be reliably predicted by various RNA folding algorithms. Double helices with consecutive Watson-Crick base-pairing may also be formed between distant RNA segments thereby facilitating long-range interactions of long-chain RNA that may be biologically functional. Here we addressed the potential formation of RNA duplex motifs by long-range RNA-RNA interactions of distantly located matching sequence elements of a single long-chain RNA.

**Results:**

We generated a Python-based software tool that identifies consecutive RNA duplex elements at any given length and nucleotide content formed by distant sequences. The software tool, dubbed *RNAslider*, is built on the theoretical RNA structure prediction algorithm Mfold. Source code and sample data sets are available on demand. We found that a small ratio of human genes including the Argonaute (Ago)-like gene family encode mRNAs containing highly GC-rich non-hairpin duplex elements (GC-helix) of equal to or more than 8 base pairs in length and we provide experimental evidence for their biological significance.

**Conclusion:**

GC-helices are observed preferentially within the 5^′^-region of mRNAs in an evolutionarily conserved fashion indicating their potential biological role. This view is supported experimentally by post-transcriptional regulation of gene expression of a fusion transcript containing 5^′^-sequences of human mRNA^Ago2^ harbouring GC-helices and down-stream coding sequences of Renilla luciferase.

## Background

Post-transcriptional regulation of gene expression and viral functions, i.e. regulation on the level of gene-specific RNAs usually involve structural and functional domains of cellular mRNAs or viral transcripts. For example, the TAR element of the human immunodeficiency virus type 1 (HIV-1), a sequence stretch of approximately 60 nucleotides within the 5^′^-region of genomic HIV-1 RNA as well as the 5^′^-portion of viral mRNAs, adopts a thermodynamically stable stem/loop structure that is functionally involved in the regulation of elongation of transcription [1]. Further, internal ribosomal entry sites, mostly found in viral sequences, form defined structural elements that are necessary for translation of protein-coding transcripts (for review see. [2,3]. On the level of cellular genes, an extra-stable local folding unit of the 5^′^-untranslated region of the murine p53 mRNA seems to be involved in post-transcriptional control of its level of protein translation [4]. In summary, structural and functional RNA modules, often located upstream of the translational initiation site or overlapping with it, are known to be involved in the regulation of protein biosynthesis. Such regulatory local RNA folding units may interact with modulators such as complementary RNA sequences in case of microRNA or antisense RNA-mediated control of gene expression (for review see: [5]), RNA-binding proteins (for review see: [6]), or low-molecular metabolites like in case of ribo-switches [7]. In most cases regulatory RNA units are composed of consecutively neighbouring sequence segments and only in rare cases long-range interactions seem to be involved in the formation of regulatory local structures like in IRES elements (e.g. [8,9]) or certain classes of catalytic RNA (e.g. [10]). Particularly simple regulatory modules of limited length such as the TAR element, the RRE element, or packaging signals of the human immunodeficiency virus type 1 (HIV-1) often adopt structural domains that coincide with local minimal energy states [11,12]. Studies on the biological role of Argonaute (Ago) genes in human cells indicate a potential post-transcriptional control step involved in regulating endogenous levels of the family member Ago2 [13]. Further, preliminary experimental studies in our laboratory indicate specific binding of RNA sequences surrounding the AUG start codon of the Ago2 mRNA with proteins involved in RNA interference (Sczakiel, unpublished data). This attracted us to study the 5^′^-UTR of mRNA^Ago2^ with regard to potential RNA cis-elements. Initial computational RNA folding studies indicated the existence of two helices formed by consecutive GC base pairs within the 5^′^-UTR and upstream coding sequences which seemed to represent a rare case. Here we studied whether the occurrence of GC-rich helices could be biologically relevant. Systematic computational and phylogenetic studies as well as experimental evidence in a mammalian cell system support the view that GC-rich duplex motifs formed by distant RNA segments, unlike typical hairpin elements, could bear biological functions.

## Methods

### Helix definitions

In this study we focused on predicted duplex elements composed of distant sequence segments and set definitions such that they could be distinguished from usual hairpin motifs. Hence we first defined two prerequisites, a minimal distance of 40 nt between two helix strands and a minimal number of 16 bp within this inter-segment region to differentiate helices from hairpin elements as it requires RNA structure at a position where a loop is found in hairpin elements. First screens indicated that these conditions result in a very small number of predicted long-range duplexes at a duplex length of greater than or equal to 8 bp. It should be noted that the software tool created in this study also accepts any other kind of duplex settings including (i) the base composition, (ii) different characteristics of the inter-segment sequence which may be longer, shorter or adopt other structures, (iii) and the number of consecutive base pairs included in the duplex of interest. For a more detailed description see Additional file 1: Table S1.

### Search for GC-rich double helices

We decided to calculate and analyse the secondary structure space rather than analysing primary sequences for inverted repeats which may form duplex elements. Thereby, we assume, false GC-helix predictions can be minimised which may increase the significance of predicted helices compared to merely location of reverse complementary GC sub-sequences. The software tool described and applied here is written in Python version 2.6.1 and, additionally, integrates the pyExcelerator library (http://pyexcelerator.sourceforge.net). The source code and a sample data set are available for academic users on request. In this study, Mfold version 2.3 was used for mRNA secondary structure prediction, additionally we used Mfold 3.4 (http://mfold.rna.albany.edu) and Sfold 2.2 (http://sfold.wadsworth.org) [14-17]. However, generally most other RNA secondary structure prediction software can be used with our tool as well. Mfold 2.3 was chosen because we found the best match for the structure-function relationship of antisense oligonucleotides (asON) and siRNA, respectively by Mfold version 2.3 versus a number of six alternative folding software tools [18]. When considering that mRNAs (polII transcripts) usually serve as target RNA for asON and siRNA one might conclude that this Mfold version predicts local structures of mRNA in a reliable fashion [19].

The computational prediction of secondary structure of RNA was based on a collection of a large number of folded sequence stretches differing in position or length or both along a given mRNA of interest. These stretches are termed ‘window’ here. Pools of folded sub-sequences can be defined systematically by sliding a window at a given step width along the sequence to be analysed. Predicted local structural elements that occur at high frequency in a large number of folded relevant windows, regardless of neighbouring sequences, turn out to have a greater chance of being meaningfully related to the biology of corresponding RNA elements. This approach of analysing RNA secondary computationally turned out to produce clearly more reliable results compared with a single fold of the complete long-chain RNA sequence of interest. Further, in terms of calculation time this concept seems to provide advantages (see paragraph 3.5).

In this study, runs with Mfold were performed with varying window sizes between 500 and 1200 nucleotides at a step width of 20 nucleotides for each mRNA sequence. Ten optimal and suboptimal structures were considered per fold. For example, at a step width of 20 nt and a window size of 800 nt a RNA sequence of 3500 nucleotides in length (appr. the length of hAgo2 mRNA) means 135 Mfold runs resulting in 1350 overlapping structures. Finally, the structures were scanned for helices using the definitions in 2.1.

### Automation of mRNA secondary structure analysis

**Figure 1:**
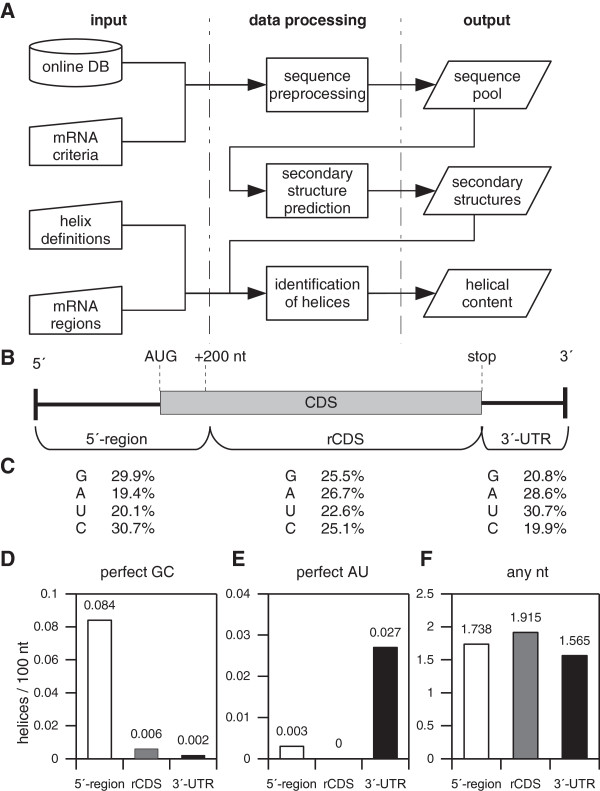
**Characterisation of nucleotide composition of long-range duplexes in mRNA.** Schematic depiction of the computational search for duplexes (**A**). Sub-regions of mRNA are defined in (**B**) and their nucleotide composition is listed in (**C**). The occurrence and nucleotide composition of long-range duplexes as a function of mRNA region is shown in (**D**-**F**).

The automated identification of duplexes composed of distant segments is illustrated as flow chart in Figure 1A. First, a pool of mRNA sequences is down-loaded from on-line resources like EMBL Nucleotide Sequence Database (http://www.ebi.ac.uk/embl/) or NCBI Nucleotide (http://www.ncbi.nlm.nih.gov/nuccore). The generated sequence pool contains mRNA species of interest for which secondary structures are predicted in a systematic fashion that has been described in detail recently [20]. Briefly, a long-chain sequence is sub-divided into overlapping sequence segments (windows) starting at position #1 and sliding along the complete sequence. Secondary structures are predicted and, depending on the step width of the shifting window and its length, a large number of differing structure predictions can be produced. This output of secondary structures is parsed for user-defined duplex motifs and is used for identification of species with specific characteristics such as GC-rich helices formed by distant sequence segments in a non-hairpin like fashion. The user is able to filter hits by setting parameters such as the base composition of the duplex, the range of distance between strands, and the range of duplex length.

### Region definition of mRNA

In this study, mRNAs were subdivided into three functional regions (see Figure 1B), the 5^′^-region which includes the 5^′^-UTR and 200 nt of the upstream coding sequences, the remaining coding region (rCDS) which spans the remaining CDS, and the 3^′^-UTR.

### Selection of human and non-human RNAs

Analyses were restricted to mRNAs with intact 5^′^-UTR, CDS, and 3^′^-UTR. Human sequences were chosen randomly from the data bases. Furthermore, we included 10 sequences, coding for members of the human Argonaute protein family. They can be subdivided into five PIWI-like (PIWI1, PIWI2 transcript variant 1, PIWI2 transcript variant 2, PIWI3, PIWI4) and five AGO-like (Ago1, Ago2 , Ago3 transcript variant 1, Ago3 transcript variant 2 , Ago4) mRNAs [21]. In addition, we investigated 49 orthologous non-human Argonaute mRNAs, which originate from the 13 organisms *Pan troglodytes, Canis lupus familiaris, Bos taurus, Oryctolagus cuniculus, Drosophila melanogaster, Mus musculus, Rattus norvegicus, Gallus gallus, Macaca mulatta, Mustel aputorius furo, Sus scrofa, Xenos puslaevis* and *Danio rerio*. Many non-human sequences found in data bases were incomplete or labelled ‘predicted’ to the time of this work and were thus excluded. However, we further included additional 12 predicted Argonaute sequences from *Pan troglodytes* and one predicted sequence from Canis lupus familiaris. Additionally, 28 non-coding RNA transcripts from *Homo sapiens* and *Mus musculus* were investigated. We also generated a pool of random sequences by re-shuffling human mRNAs while conserving the nucleotide frequencies of their 5'-UTRs, CDS’s, and 3^′^-UTRs. All sequences used in this study are listed in Additional file 1: Table S2.

### Evolutionary conservation

During comparative analyses the structure of paralogs and orthologs of hAgo2 mRNA were compared. Their duplex content was characterised with respect to (i) frequency, (ii) location within RNA sequences, (iii) duplex length, (iv) nucleotide composition, and (v) structure of inter-segment sequences.

## Results and discussion

### Double helices composed of distant RNA segments

Although long RNA strands may be extensively folded, consecutively base-paired double helices longer than 4 to 6 bp located outside of hairpin elements (including short helices which are being packed together in the overall architecture of RNA) occur rarely in resolved and predicted folding units [22]. We studied the occurrence of non-hairpin duplex elements including GC-rich or AU-rich helices in a library of human mRNA sequences by using this software tool. We consider the library of human RNA sequences as representative because they were chosen randomly and include mRNA from transcription factors, metabolic regulators, receptors, enzymes, ion channels, carrier proteins, glycol-proteins, structural proteins, heat shock proteins as well as yet uncharacterised cDNA sequences. Firstly, we retrieved sequences matching the above criteria (Additional file 1: Table S2A) and performed secondary structure predictions as described in Methods (2.3) using a window size of 800 nt and a step width of 20 nt. Finally, the results were screened for duplex elements, i.e. helices according to the definitions described in 2.1. Human mRNAs vary in total length which is also true for their domains, i.e. the 5^′^-UTR, CDS, and 3^′^-UTR. To account for these variations in length, we describe the helix frequency per 100 nt.

When non-hairpin duplexes composed of 8 or more consecutive base pairs were considered, a frequency of 1.774 duplexes per 100 nt was found in the examined mRNA population. In 21 of the evaluated structures (32%) one or more GC-helices were found. The overall GC-helix frequency was 0.01 helices / 100 nt. In comparison, AU-helices occur at about the same frequency and were found in 23 of the scanned structures (35%). In summary, the sequences analysed in this study showed an average length of non-hairpin double helices of 9 ±1.5 bp (S.D).

For GC-helices an average distance between the two complementary strand segments of 180 ± 177 nt was observed. For AU-helices this value was 231 ± 181 nt and for all helices regardless of base composition we found 327.63 ± 227 nt. These characteristics were similar between human and non-human mRNAs. The highest degree of consistency at the inter-species comparison was seen in the 5^′^-region, which by itself is very rich in GC-helices (compare 3.3). For AU-helices this agreement is less pronounced.

Non-coding RNA transcripts and shuffled human mRNAs were taken as two separate control groups to test whether the abundance of predicted GC- and AU-duplexes in the examined mRNA library was non-random. No GC-duplexes of 8 or more consecutive base pairs were found in any examined non-coding RNA transcript. The GC-helix frequency in the shuffled mRNAs was 0.002 and thus lower by a factor of 5 compared to the human mRNA population. In contrast, AU-duplexes were more abundant in non-coding RNA compared to human mRNA. While their frequency in shuffled mRNA sequences was in the same magnitude as in the mRNA population. Please refer to Additional file 1: Table S4 for details.

### Helix location within the mRNA sequence

Next we wondered whether GC-helices are equally distributed along mRNA which we sub-divided into three domains (Figure 1B and Methods 2.4). The nucleotide composition of these domains of the human mRNA sequences studied here is summarised in Figure 1C. Systematic computational analyses of the human mRNA sequences and their predicted structures show that GC-helices predominantly occur within the 5^′^-region of mRNAs (0.084 per 100 nt versus 0.006 in the rCDS and 0.002 in the 3^′^-UTR, respectively) while AU-helix frequency is much lower and AU-helices predominantly occur within the 3^′^-UTR (Figure 1D and E). The distribution of predicted duplex elements within mRNAs can be found in Additional file 2 and a detailed listing of all predicted GC-helices in relation to the start codon is provided in Additional file 3. In contrast, the occurrence of duplex elements with mixed base compositions seems to be more balanced between the three regions (1.738, 1.915 and 1.565 per 100 nt for 5^′^-region, rCDS, and 3^′^-UTR, respectively; Figure 1F). Replacing the 5^′^-region by 5^′^-UTR and hence rCDS by CDS (i.e. the complete coding region) does not alter these figures substantially. Likewise, the distribution of GC-rich helices is similar to that of classical GC-rich stem loops (data not shown). The uneven distribution of GC- and AU-helices between the three defined regions might be related to a certain extent to the base composition of these regions (Figure 1C) but it seems to be unlikely that this fully explains the sharp differences of their local occurrence. To test this, we searched for GC- and AU-duplex motifs in randomly shuffled RNA sequences. Here, GC-duplex frequencies are lower than found in the mRNA population and their location within the three sequence domains is balanced in the pool of shuffled mRNA. The preferred occurrence of GC duplexes in the 5^′^-region of mRNAs was not be observed in case of shuffled mRNAs (Additional file 1: Table S4). Also AU-duplexes occur in a balanced fashion within the three sequence domains in contrast to the mRNA sequences, where AU-duplexes are preferably located in the 3^′^-UTR. It should be mentioned that the position of GC-rich helices does not coincide with CpG islands on the level of chromosomal DNA. In the following we focused on double helices predominantly or exclusively composed of GC base pairs.

### GC-helix abundance and gene family

Conserved RNA structure elements may be involved in biological functions [23]. Structural and related functional conservation [24,25] can be supported by comparative analysis between classes of genes and by studying evolutionary conservation. Here it became obvious that GC-helices occur in the 5^′^-UTR regions of the three human AGO-like mRNAs Ago1, Ago2, and Ago4 while being completely absent in human PIWI-like mRNAs. This finding is also true for other vertebrates studied here. Hence, the occurrence of GC-helices and their location within the 5^′^-UTR of mRNAs of the Argonaute gene family might be biologically meaningful.

### Phylogenetic analysis of GC-helices in argonautes

The accumulation of GC-helices was further studied for orthologous sequences. This study was restricted to 49 sequences from 13 different organisms (Additional file 1: Table S2) since many non-human sequences found in on-line databases were not suited for our study because of limitations described in the Methods section. Out of 49 orthologous Argonaute sequences, 10 were GC-helix positive. The ten hits share two criteria: they are of vertebrate origin and their gene class is AGO-like Argonautes: *Bos taurus* Ago1 and Ago2 (two variants), *Mus musculus* Ago2 (two variants) and Ago4, *Rattus norvegicus* Ago4, *Pan troglodytes* two Ago-like (Transcriptome Shotgun Assembly), as well as, *Gallus gallus* Ago4. As there exist only few complete non-human Argonaute mRNA sequence files, we additionally evaluated 13 predicted sequences. However, since they were not determined experimentally, but are estimated from homologue sequences by GNOMON (http://www.ncbi.nlm.nih.gov/projects/genome/guide/gnomon.shtml), we separated these results from the rest. GC-helices were found in four out of twelve 5^′^-UTR-regions of predicted AGO-like mRNAs in *Pan troglodytes*.

### Compatibility with different secondary structure prediction algorithms

Compared to the sliding window approach based on Mfold 2.3 (as described in Paragraph 2.2), Sfold 2.2 and Mfold 3.4 predict 48% and 31% less GC-duplex motifs of 8 or more base pairs in the investigated mRNA library, respectively (compare Additional file 1: Table S4). However, the qualitative finding of this study is independent of the kind of secondary structure prediction algorithm. All three compared algorithms predict that 5^′^-regions of human and non-human mRNAs are rich in GC-helices compared to rCDS’s and 3^′^-UTRs, while AU-helices are most abundant in 3^′^-UTRs.

In technical terms, on a PC with Intel(R) Core(TM) 2 Duo CPU E4500 @ 2.20GHz and 2 GB memory, running openSUSE 11.1 (×86 64) folding of a 9000 nt sequence by Mfold 2.3 takes on average 206 min. At a step width of 20 nt and a window size of 800 nt a RNA sequence of 9000 nucleotides in length means 410 Mfold runs which all together take about 60 min on the same system. This is in accordance with the fact that the underlying Mfold algorithm computes in time proportional to the cube of the folded length of sequence. Because Mfold 3.4 and Sfold 2.2 computations were performed on on-line servers, we cannot directly compare their performance. However, in case of Sfold 2.2 calculations of 9000 nt sequences took several days (mainly due to queuing) and in case of Mfold 3.4 the results were ready for down-load after 43 min.

### A GC-helix within the 5^′^-region of the Ago2 mRNA is involved in post-transcriptional regulation of Ago2 gene expression

**Figure 2:**
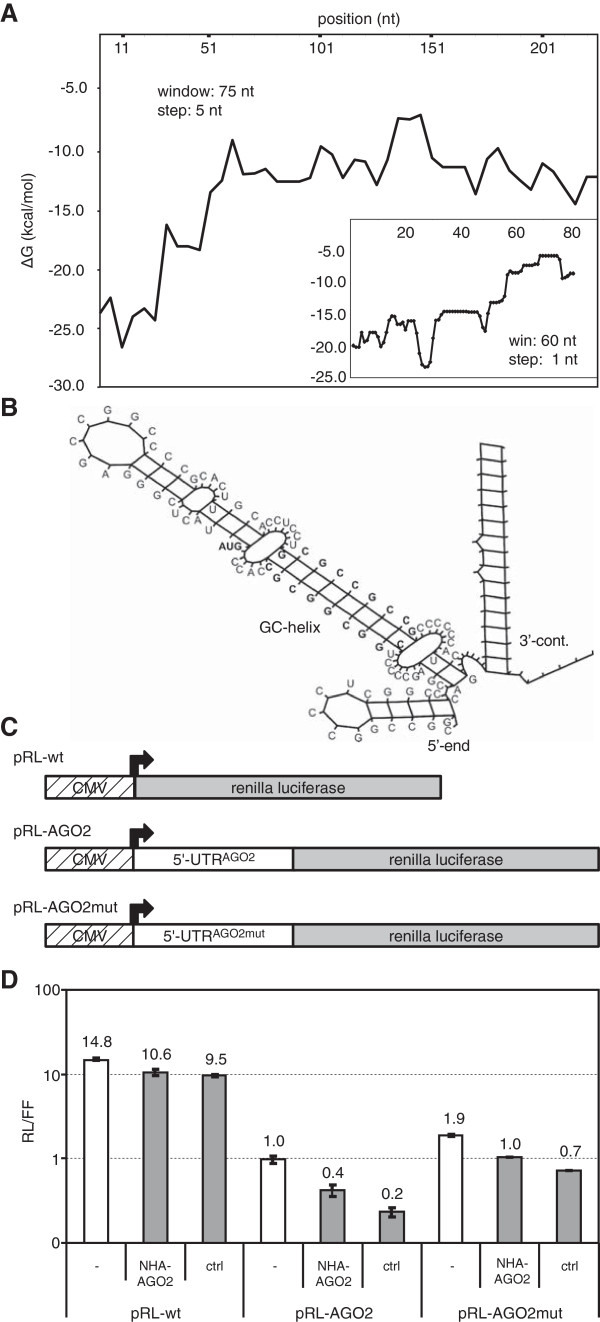
**Structural and functional analysis of the 5**^**′**^**-region**^**Ago2**^**.** The folding potential (**A**) and predicted 2D structure of the 5^′^-region of mRNA^Ago2^ (**B**) suggest two GC-helices. A luciferase gene containing the mRNA^Ago2^ 5^′^-region (pRL-Ago2) but not a derivative mutated in the GC-helix region nor the wt gene (**C** and **D**) indicate Ago2-mediated up-regulation of gene expression (NHA-Ago2).

In order to shed more light on the existence of the predicted GC-helix in the 5^′^-region of mRNA^Ago2^ we calculated the local folding potential, a parameter that is often correlated with biologically functional and stably folded domains [19,26]. The local energy minimum related to the folding unit between positions 20 and 80 (minimum at position 20 at a window size of 60 nt; (Figure 2A) coincides with the 9 bp GC-helix depicted in Figure 2B. In order to test whether this GC-helix could be involved in regulation of Ago2 gene expression in functional terms we studied the role of this element in gene expression studies in cell culture as well as its characteristics in protein binding studies *in vitro*.

Firstly, we cloned recombinant plasmids containing the Renilla luciferase open reading frame fused downstream to the 5^′^-UTR of mRNA^Ago2^ termed pRL-Ago2 (Figure 2C and Additional file 1: Table S3). For control purposes we used the parental luciferase-harbouring plasmid (pRL) or a variant of pRL-Ago2 in which the GC-helices were destroyed by nucleotide exchanges (pRL-Ago2mut, Additional file 1: Table S3). In order to study possible effects of over-expression of Ago2 on the 5^′^-UTR^Ago2^-luciferase fusion mRNA we co-transfected either of these plasmids together with an established recombinant eukaryotic expression plasmid for Ago2 (pNHA-Ago2; [27]) into human ECV304 cells. The results shown in Figure 2D indicate an up-regulation of the 5^′^-UTR^Ago2^-luciferase fusion by over-expressed Ago2 protein but not in the presence of the parental Ago2-negative plasmid termed ‘contr’. Further, basal expression levels of the 5^′^-UTR^Ago2^-luciferase fusion seem to be higher than the levels of controls (Figure 2D, open bars) which is consistent, to a certain extent, with the assumption that endogenous Ago2 increases its expression via the 5^′^-UTR^Ago2^.

Secondly, we performed binding studies with HeLa cell extracts and *in vitro* transcribed Ago2 mRNA 5^′^-sequences or control sequences depicted in Figure 2C & D. These studies indicate increased binding of proteins involved in RNA interference including Ago2 itself with the GC duplex-containing sequences of Ago2 mRNA. This observation is consistent with the involvement of the GC duplex of the Ago2 mRNA in post-transcriptional control of gene expression (Sczakiel, unpublished data).

## Conclusion

This study provides a computational tool to search for duplex RNA elements formed by distantly located segments (≥ 40 nt) of complementary RNA in a non-hairpin fashion. Phylogenetic analyses indicate a non-random occurrence of these structural elements dependent on the base composition of the helical strands in mRNAs transcribed from certain genes and gene families. In conjunction with initial biological observations our findings indicate a potential role of GC-rich helices for post-transcriptional regulatory processes within the mRNAs of the Argonaute gene family.

## Competing interests

The authors declare that they have no competing interests.

## Authors’ contributions

SD did programming, compiled and calculated data used here, and interpreted results together with GS. GS interpreted results in the light of the biology of post-transcriptional regulation and both authors prepared this manuscript. Both authors read and approved the final manuscript.

## Supplementary Material

Additional file 1: Tables S1-S4 **Table S1:** Parameters for duplex definition. **Table S2:** RNA sequences used in this study. **Table S3:** Sequences of pRL-Ago2 and pRL-Ago2mut plasmids. **Table S4:** Distribution of GC- and AU-duplexes as predicted by three different algorithms. Click here for file

Additional file 2 **Characterisation of mRNA sequences.** In this data file mRNA strands are divided into three regions, (**a**) 5^′^-region (5^′^-UTR + 200 nt), (**b**) the remaining coding region (rCDS), and (c) the 3^′^-UTR. Additionally, rCDS and 3^′^-UTR can be combined to region (**d**). All numbers for region definitions are given in nt. For each region and for each mRNA motif frequencies are provided in absolute numbers (no.) and in frequency per 100 nt (freq.). Data is shown for four predicted secondary structure motifs, AU-helix, GC-helix, GC-stem, helix in general (≥ 8 uninterrupted base pairs, strand distance ≥ 40 nt, ≥ 16 nt in linker region involved in base pairings). Their exact definitions are provided in Additional file 1: Table S1. Click here for file

Additional file 3 **Predicted duplex elements and their locations within mRNAs.** This file lists predicted GC-helices (≥ 8 uninterrupted GC pairs, strand distance ≥ 40 nt, ≥ 16 nt in linker region involved in base pairings) of human and non-human mRNAs investigated in this study. Information includes the sequences of the two complementary helix strands, their positions within mRNA sequence, frequency of the motif in predicted structures (in motifs / 100 nt), distance from start codon. Click here for file
