# Supplementary material for: Computational identification of biologically functional non-hairpin GC-helices in human Argonaute mRNA
Source: BMC Bioinformatics. 2013 Apr 10;14:122. doi: 10.1186/1471-2105-14-122 (PMC3626786; doi:10.1186/1471-2105-14-122)
Supplement: Additional file 1: Tables S1-S4 — Table S1: Parameters for duplex definition. Table S2: RNA sequences used in this study. Table S3: Sequences of pRL-Ago2 and pRL-Ago2mut plasmids. Table S4: Distribution of GC- and AU-duplexes as predicted by three different algorithms. [file 1471-2105-14-122-S1.pdf]

Supplementary Data

Table S1: Parameters for duplex definition. The software tool provides seven free parameters to specify duplex classes. It is possible to restrict base composition, different characteristics of the inter-segment sequence which may be longer, shorter or adopt other structures, and the number of consecutive base pairs with the duplex of interest. Columns 1-3 describe these parameters; columns 4-11 define duplex classes as used in this study.

parameter	description	co-domain	duplex	helix	stem	AU-duplex	AU-helix	GC-duplex	GC-helix	GC-stem
min_dist	minimal length of the inter-segment sequence	$\mathbb{N}^0$ (nt)	0	40	ULTD	0	40	0	40	0
min_linker_pair	minimal number of bp in inter-segment sequence	$\mathbb{N}^0$ (bp)	0	6	0	0	6	0	6	0
max_dist	maximal length of the inter-segment sequence	$\mathbb{N}^0$ (nt)	ULTD	ULTD	39	ULTD	ULTD	ULTD	ULTD	39
max_linker_pair	maximal number of bp in inter-segment sequence	$\mathbb{N}^0$ (bp)	ULTD	ULTD	5	ULTD	ULTD	ULTD	ULTD	5
min_len	minimal number of consecutive bp with the duplex of interest	$\mathbb{N}$ (bp)	8	8	8	8	8	8	8	8
anti_parallel	whether duplex formation between anti-parallel strands is valid	Boolean	FALSE	FALSE	FALSE	FALSE	FALSE	FALSE	FALSE	FALSE
max_AU	maximal number of AU bp allowed in duplex	$\mathbb{N}^0$ (nt)	ULTD	ULTD	ULTD	ULTD	ULTD	0	0	0
max_GU	maximal number of GU bp allowed in duplex	$\mathbb{N}^0$ (nt)	ULTD	ULTD	ULTD	0	0	0	0	0
max_GC	maximal number of GC bp allowed in duplex	$\mathbb{N}^0$ (nt)	ULTD	ULTD	ULTD	0	0	ULTD	ULTD	ULTD

Table S2: List of mRNA sequences included in the study. **A** We restricted to mRNAs with intact 3´-UTR, CDS, and 5´-UTR. 77 sequences of human origin, inclusive 10 mRNAs of the Argonaute gene family. **B** 49 sequences of non-human Argonaute orthologs plus 13 predicted ones. **C** 28 non-coding RNA transcripts from *Homo sapiens* and *Mus musculus*. All sequences are freely available online at resources like EMBL Nucleotide Sequence Database (http://www.ebi.ac.uk/embl/) and NCBI Nucleotide (http://www.ncbi.nlm.nih.gov/nuccore).

A

name	Accession
ACIN1	NM_014977
ALDH7A1	NM_001182
API5-1	NM_001142930
APP-2	NM_201413
ATG13-1	NM_001142673
ATP1A1-4	NM_001160234
BCKDHB-1	NM_183050
CCDC120-1	NM_001163321
CD36	NM_001001548
COL2A-1	NM_001844
COX2	NM_000963
CP-2	NM_000096
DMD-Dp71b	NM_004016
E2F3	NM_001949
FGFR2-7	NM_001144917
FGFR3-1	NM_000142
GRM7-1	NM_000844
HIV-1-pNL4-3	AF324493
ICAM1	P05362 (SwissProt)
INPPL1	NM_001567
IPO13	NM_014652
ITG-AV	NM_002210
ITGAM	NM_001145808

name	Accession
SAPS3-2	NM_001164163
SCIDA-2	NM_022487
SELE	XM_057446
SLC25A12	NM_003705
SLC6A4	NM+AF8-001045
SLCO2B1	NM_007256
STOX2	BC146754
STXBP1-1	NM_003165
TCF7L2-2	NM_030756
TIAL1-2	NM_001033925
TMEM2	BC146780
TRP3-1	NM_001130698
UNC51-2	NM_001142610
USP19	BC146752
WFS1	NM_001145853
ZNF828	NM_001164145
hAGO1 (EIF2C1)	NM_012199
hAGO2 (EIF2C2)	NM_012154
hAGO3-1 (EIF2C3)	NM_024852
hAGO3-2 (EIF2C3)	NM_177422
hAGO4 (EIF2C4)	NM_017629
phosphodiesterase	L20971
PIWIL1 (HIWI)	NM_004764

name	Accession
KTN-1	NM_182926
LAMB3	NM_001127641
LAP2-1	NM_001017963
MANEA	BC146671
MYB	M15024
MYB-1	NM_001130173
NHE5	NM_004594
NOS3-1	NM_000603
PGY1	M14758
PLOD2	NM_000935
PRKD1	NM_002742
PROM1	NM_006017
RAD4-2	NM_001145769
RAF1	XM_051583
Rab11-FIP3-1	NM_014700

name	Accession
PIWIL2-1 (HILI-1)	NM_001135721
PIWIL2-2 (HILI-2)	NM_018068
PIWIL3	NM_001008496
PIWIL4 (HIWI2, HILI2)	NM_152431
DCR1-T1	NM_030621
DCR1-T2	NM_177438
RNASEN-T1	NM_013235
RNASEN-T2	NM_001100412
TP53-ISOA-T1	NM_000546
TP53-ISOA-T2	NM_001126112
TP53-ISOB-T3	NM_001126114
TP53-ISOC-T4	NM_001126113
TP53-ISOD-T5	NM_001126115
TP53-ISOE-T6	NM_001126116
TP53-ISOF-T7	NM_001126117

B

name	origin	Accession
Ago3b	Danio rerio	NM_001160028
Ago4	Danio rerio	NM_001252559
ZILI (piwil2)	Danio rerio	NM_001080199
ZIWI (piwil1)	Danio rerio	NM_183338
AGO1	Rattus norvegicus	NM_001191765
AGO2	Rattus norvegicus	NM_001271193
AGO4 (Eif2c4)	Rattus norvegicus	NM_001106686
PIWIL1	Rattus norvegicus	NM_001108853
PIWIL2-2	Rattus norvegicus	NM_001107276

name	origin	Accession
MILI (PIWIL2)	Mus musculus	NM_021308
MIWI (PIWIL1)	Mus musculus	NM_021311
MIWI2 (PIWIL4)	Mus musculus	AB258534
AGO1clone (Eif2c1, meIF2C1)	Mus musculus	AK080954
AGO2 (Eif2c2, GERp95, meIF2C2)	Mus musculus	NM_153178
AGO2 (alternative)	Mus musculus	NM_153178
AGO3 (Eif2c3)	Mus musculus	NM_153402
AGO4 (Eif2c4)	Mus musculus	NM_153177
AGO1-A	Drosophila melanogaster	NM_166020
AGO1-B	Drosophila melanogaster	NM_079010
AGO1-C	Drosophila melanogaster	NM_166021
AGO2-B	Drosophila melanogaster	NM_140518
AGO2-C	Drosophila melanogaster	NM_168626
AGO2-C (alternative)	Drosophila melanogaster	NM_168626
AGO2-E	Drosophila melanogaster	NM_001274953
AGO3-D	Drosophila melanogaster	NM_001043162
AGO3-E	Drosophila melanogaster	NM_001043164
AGO3-F	Drosophila melanogaster	NM_001043163
AUB-A	Drosophila melanogaster	NM_057386
AUB-C	Drosophila melanogaster	NM_001103674
PIWI	Drosophila melanogaster	NM_057527
AGO2 (EIF2C2)	Oryctolagus cuniculus	NM_001082710
PIWIL2-t2 (predicted)	Canis lupus familiaris	XM_853957
EIF2C1-t1 (predicted)	Pan troglodytes	XM_001167251
Ago (TSA)	Pan troglodytes	GABF01000457
Ago (TSA)	Pan troglodytes	GABD01009992
EIF2C1-t2 (predicted)	Pan troglodytes	XM_001167312

name	origin	Accession
EIF2C1-t3 (predicted)	Pan troglodytes	XM_001167349
EIF2C1-t4 (predicted)	Pan troglodytes	XM_513312
EIF2C2-t1 (predicted)	Pan troglodytes	XM_001142838
EIF2C2-t2 (predicted)	Pan troglodytes	XM_001143064
EIF2C3-t1 (predicted)	Pan troglodytes	XM_001167488
EIF2C3-t2 (predicted)	Pan troglodytes	XM_001167515
EIF2C3-t3 (predicted)	Pan troglodytes	XM_001167651
EIF2C3-t4 (predicted)	Pan troglodytes	XM_001167675
EIF2C3-t5 (predicted)	Pan troglodytes	XM_524664
Ago1	*Bos taurus*	NM_001205899
Ago2	*Bos taurus*	BT030588
Ago2 (alternative)	*Bos taurus*	BC151491
Ago3	*Gallus gallus*	NM_001030900
Ago4	*Gallus gallus*	NM_001039276
Ago (TSA)	Macaca mulatta	JU475189
Ago (TSA)	Mustela putorius furo	JP009204
Ago2	Oryctolagus cuniculus	NM_001082710
Ago1	*Sus scrofa*	NM_001194976
Ago2	*Sus scrofa*	NM_001194975
Ago3	*Sus scrofa*	NM_001194974
Ago4	*Sus scrofa*	NM_001194972
Ago1 (clone)	*Xenopus laevis*	BC077863
Ago2	*Xenopus laevis*	NM_001093519
Ago2 (clone)	*Xenopus laevis*	CR761661
Ago4	*Xenopus laevis*	NM_001096105

C

name	origin	Accession
HOTAIR-t1	Homo sapiens	NR_047517
HOTAIR-t2	Homo sapiens	NR_003716
JPX	Homo sapiens	NR_024582
PCAT1	Homo sapiens	HQ605084
PCAT14	Homo sapiens	HQ605085
SUZ12	Homo sapiens	NM_015355
Tsix	Homo sapiens	NR_003255
lincRNA-ST8SIA3	Homo sapiens	HQ315778
intergenic non-protein coding RNA	Homo sapiens	JX088243
Tsix	Homo sapiens	NR_002844
Xist-t1	Mus musculus	NR_001463
Xist-t2	Mus musculus	NR_001570
AIR-t1	Mus musculus	DQ275619
AIR-t1b	Mus musculus	DQ275617
AIR-t2	Mus musculus	DQ275620
AIR-t3	Mus musculus	DQ275618
lgf2r-as-Airn-t1	Mus musculus	NR_027773
lgf2r-as-Airn-t2	Mus musculus	NR_027772
lgf2r-as-Airn-t3	Mus musculus	DQ220013
lgf2r-as-Airn-t4	Mus musculus	NR_027784
lgf2r-as-t1	Mus musculus	DQ220010
lgf2r-as-t2	Mus musculus	DQ220012
lgf2r-as-t3	Mus musculus	NR_002853
lgf2r-as-t4	Mus musculus	DQ220011
lgf2r-as-t5	Mus musculus	DQ220014
Xist	*Bos taurus*	NR_001464

Table S3: Plasmid sequences of pRL-AGO2 and pRL-AGO2mut.

colour coding:

blue	CMV promoter
brown	TATA-Box / start of transcription for CMV
black underlined	cloning site SacI/NheI
blue underlined	sequence primer
red	Ago2 (244nt)
red underlined	mutations Ago2
green	AUG Ago2
yellow	ORF Renilla-Luciferase
pink	glycin linker

> pRL-AGO2 (pRL-CMV_Ago2_244_SacI_NheI)

```
AGATCTTCAATATTGGCCATTAGCCATATTATTCATTGGTTATATAGCATAAATCAATATTGGCTATTGGCCATTGCATACGTTGTATCTATATCATAATATGTACATTTATATTGGCTCATGTCCAAT
ATGACCGCCATGTTGGCATTGATTATTGACTAGTTATTAATAGTAATCAATTACGGGGTCATTAGTTCATAGCCCATATATGGAGTTCCGCGTTACATAACTTACGGTAAATGGCCCGCCTGGCTGACC
GCCCAACGACCCCCGCCCATTGACGTCAATAATGACGTATGTTCCCATAGTAACGCCAATAGGGACTTTCCATTGACGTCAATGGGTGGAGTATTTACGGTAAACTGCCCACTTGGCAGTACATCAAGT
GTATCATATGCCAAGTCCGCCCCCTATTGACGTCAATGACGGTAAATGGCCCGCCTGGCATTATGCCCAGTACATGACCTTACGGGACTTTCCTACTTGGCAGTACATCTACGTATTAGTCATCGCTAT
TACCATGGTGATGCGGTTTTGGCAGTACACCAATGGGCGTGGATAGCGGTTTGACTCACGGGGATTTCCAAGTCTCCACCCCATTGACGTCAATGGGAGTTTGTTTTGGCACCAAAATCAACGGGACTT
TCCAAAATGTCGTAATAACCCCGCCCCGTTGACGCAAATGGGCGGTAGGCGTGTACGGTGGGAGGTCTATATAAGCAgagctcGTTTAGTGAACCGGGCCGCCCCTCGGCCCCGGAGCCCCTCGGCGGC
GCCACCATGTACTCGGGAGCCGGCCCCGCACTTGCACCTCCTGCGCCGCCGCCCCCCATCCAAGGATATGCCTTCAAGCCTCCACCTAGACCCGACTTTGGGACCTCCGGGAGAACAATCAAATTACAG
GCCAATTTCTTCGAAATGGACATCCCCAAAATTGACATCTATCATTATGAATTGGATATCAAGCCAGAGAAGTGCCCGAGGggtggcggaggtggcggattgctagcCACCATGACTTCGAAAGTTTAT
GATCCAGAACAAAGGAAACGGATGATAACTGGTCCGCAGTGGTGGGCCAGATGTAAACAAATGAATGTTCTTGATTCATTTATTAATTATTATGATTCAGAAAAACATGCAGAAAATGCTGTTATTTTT
TTACATGGTAACGCGGCCTCTTCTTATTTATGGCGACATGTTGTGCCACATATTGAGCCAGTAGCGCGGTGTATTATACCAGACCTTATTGGTATGGGCAAATCAGGCAAATCTGGTAATGGTTCTTAT
AGGTTACTTGATCATTACAAATATCTTACTGCATGGTTTGAACTTCTTAATTTACCAAAGAAGATCATTTTTGTCGGCCATGATTGGGGTGCTTGTTTGGCATTTCATTATAGCTATGAGCATCAAGAT
AAGATCAAAGCAATAGTTCACGCTGAAAGTGTAGTAGATGTGATTGAATCATGGGATGAATGGCCTGATATTGAAGAAGATATTGCGTTGATCAAATCTGAAGAAGGAGAAAAAATGGTTTTGGAGAAT
AACTTCTTCGTGGAAACCATGTTGCCATCAAAAATCATGAGAAAGTTAGAACCAGAAGAATTTGCAGCATATCTTGAACCATTCAAAGAGAAAGGTGAAGTTCGTCGTCCAACATTATCATGGCCTCGT
GAAATCCCGTTAGTAAAAGGTGGTAAACCTGACGTTGTACAAATTGTTAGGAATTATAATGCTTATCTACGTGCAAGTGATGATTTACCAAAAATGTTTATTGAATCGGACCCAGGATTCTTTTCCAAT
GCTATTGTTGAAGGTGCCAAGAAGTTTCCTAATACTGAATTTGTCAAAGTAAAAGGTCTTCATTTTTCGCAAGAAGATGCACCTGATGAAATGGGAAAATATATCAAATCGTTCGTTGAGCGAGTTCTC
AAAAATGAACAATAATTCTAGAGCGGCCGCTTCGAGCAGACATGATAAGATACATTGATGAGTTTGGACAAACCACAACTAGAATGCAGTGAAAAAAATGCTTTATTTGTGAAATTTGTGATGCTATTG
CTTTATTTGTAACCATTATAAGCTGCAATAAACAAGTTAACAACAACAATTGCATTCATTTTATGTTTCAGGTTCAGGGGGAGGTGTGGGAGGTTTTTTAAAGCAAGTAAAACCTCTACAAATGTGGTA
AAATCGATAAGGATCCAGGTGGCACTTTTCGGGGAAATGTGCGCGGAACCCCTATTTGTTTATTTTTCTAAATACATTCAAATATGTATCCGCTCATGAGACAATAACCCTGATAAATGCTTCAATAAT
ATTGAAAAAGGAAGAGTATGAGTATTCAACATTTCCGTGTCGCCCTTATTCCCTTTTTTGCGGCATTTTGCCTTCCTGTTTTTGCTCACCCAGAAACGCTGGTGAAAGTAAAAGATGCTGAAGATCAGT
TGGGTGCACGAGTGGGTTACATCGAACTGGATCTCAACAGCGGTAAGATCCTTGAGAGTTTTCGCCCCGAAGAACGTTTTCCAATGATGAGCACTTTTAAAGTTCTGCTATGTGGCGCGGTATTATCCC
GTATTGACGCCGGGCAAGAGCAACTCGGTCGCCGCATACACTATTCTCAGAATGACTTGGTTGAGTACTCACCAGTCACAGAAAAGCATCTTACGGATGGCATGACAGTAAGAGAATTATGCAGTGCTG
CCATAACCATGAGTGATAACACTGCGGCCAACTTACTTCTGACAACGATCGGAGGACCGAAGGAGCTAACCGCTTTTTTGCACAACATGGGGGATCATGTAACTCGCCTTGATCGTTGGGAACCGGAGC
TGAATGAAGCCATACCAAACGACGAGCGTGACACCACGATGCCTGTAGCAATGGCAACAACGTTGCGCAAACTATTAACTGGCGAACTACTTACTCTAGCTTCCCGGCAACAATTAATAGACTGGATGG
AGGCGGATAAAGTTGCAGGACCACTTCTGCGCTCGGCCCTTCCGGCTGGCTGGTTTATTGCTGATAAATCTGGAGCCGGTGAGCGTGGGTCTCGCGGTATCATTGCAGCACTGGGGCCAGATGGTAAGC
CCTCCCGTATCGTAGTTATCTACACGACGGGGAGTCAGGCAACTATGGATGAACGAAATAGACAGATCGCTGAGATAGGTGCCTCACTGATTAAGCATTGGTAACTGTCAGACCAAGTTTACTCATATA
TACTTTAGATTGATTTAAAACTTCATTTTTAATTTAAAAGGATCTAGGTGAAGATCCTTTTTGATAATCTCATGACCAAAATCCCTTAACGTGAGTTTTCGTTCCACTGAGCGTCAGACCCCGTAGAAA
AGATCAAAGGATCTTCTTGAGATCCTTTTTTTCTGCGCGTAATCTGCTGCTTGCAAACAAAAAAACCACCGCTACCAGCGGTGGTTTGTTTGCCGGATCAAGAGCTACCAACTCTTTTTCCGAAGGTAA
CTGGCTTCAGCAGAGCGCAGATACCAAATACTGTTCTTCTAGTGTAGCCGTAGTTAGGCCACCACTTCAAGAACTCTGTAGCACCGCCTACATACCTCGCTCTGCTAATCCTGTTACCAGTGGCTGCTG
```

CCAGTGGCGATAAGTCGTGTCTTACCGGGTTGGACTCAAGACGATAGTTACCGGATAAGGCGCAGCGGTCGGGCTGAACGGGGGGTTCGTGCACACAGCCCAGCTTGGAGCGAACGACCTACACCGAAC
TGAGATACCTACAGCGTGAGCTATGAGAAAGCGCCACGCTTCCCGAAGGGAGAAAGGCGGACAGGTATCCGGTAAGCGGCAGGGTCGGAACAGGAGAGCGCACGAGGGAGCTTCCAGGGGGAAACGCCT
GGTATCTTTATAGTCCTGTCGGGTTTCGCCACCTCTGACTTGAGCGTCGATTTTTGTGATGCTCGTCAGGGGGGCGGAGCCTATGGAAAAACGCCAGCAACGCGGCCTTTTTACGGTTCCTGGCCTTTT
GCTGGCCTTTTGCTCACATGGCTCGAC

> pRL-AGO2mut (pRL-CMV_Ago2_244_SacI_NheI_D1/D2mut)
AGATCTTCAATATTGGCCATTAGCCATATTATTCATTGGTTATATAGCATAAATCAATATTGGCTATTGGCCATTGCATACGTTGTATCTATATCATAATATGTACATTTATATTGGCTCATGTCCAAT
ATGACCGCCATGTTGGCATTGATTATTGACTAGTTATTAATAGTAATCAATTACGGGGTCATTAGTTCATAGCCCATATATGGAGTTCCGCGTTACATAACTTACGGTAAATGGCCCGCCTGGCTGACC
GCCCAACGACCCCCGCCCATTGACGTCAATAATGACGTATGTTCCCATAGTAACGCCAATAGGGACTTTCCATTGACGTCAATGGGTGGAGTATTTACGGTAAACTGCCCACTTGGCAGTACATCAAGT
GTATCATATGCCAAGTCCGCCCCCTATTGACGTCAATGACGGTAAATGGCCCGCCTGGCATTATGCCCAGTACATGACCTTACGGGACTTTCCTACTTGGCAGTACATCTACGTATTAGTCATCGCTAT
TACCATGGTGATGCGGTTTTGGCAGTACACCAATGGGCGTGGATAGCGGTTTGACTCACGGGGATTTCCAAGTCTCCACCCCATTGACGTCAATGGGAGTTTGTTTTGGCACCAAAATCAACGGGACTT
TCCAAAATGTCGTAATAACCCCGCCCCGTTGACGCAAATGGGCGGTAGGCGTGTACGGTGGGAGGTCTATATAAGCAgagctcGTTTAGTGAACCGAAAAGCCCCTCGGCCCCGGAGCCCCTAAAAGGC
GCCACCATGTACTCGGGAGCCGGCCCCGCACTTGCACCTCCTGCGCCGCCGCCCCCCATCCAAGGATATGCCTTCAAGCCTCCACCTAGACCCGACTTTGGGACCTCCGGGAGAACAATCAAATTACAG
GCCAATTTCTTCGAAATGGACATCCCCAAAATTGACATCTATCATTATGAATTGGATATCAAGCCAGAGAAGTGCCCGAGGggtggcggaggtggcggattgctagcCACCATGACTTCGAAAGTTTAT
GATCCAGAACAAAGGAAACGGATGATAACTGGTCCGCAGTGGTGGGCCAGATGTAAACAAATGAATGTTCTTGATTCATTTATTAATTATTATGATTCAGAAAAACATGCAGAAAATGCTGTTATTTTT
TTACATGGTAACGCGGCCTCTTCTTATTTATGGCGACATGTTGTGCCACATATTGAGCCAGTAGCGCGGTGTATTATACCAGACCTTATTGGTATGGGCAAATCAGGCAAATCTGGTAATGGTTCTTAT
AGGTTACTTGATCATTACAAATATCTTACTGCATGGTTTGAACTTCTTAATTTACCAAAGAAGATCATTTTTGTCGGCCATGATTGGGGTGCTTGTTTGGCATTTCATTATAGCTATGAGCATCAAGAT
AAGATCAAAGCAATAGTTCACGCTGAAAGTGTAGTAGATGTGATTGAATCATGGGATGAATGGCCTGATATTGAAGAAGATATTGCGTTGATCAAATCTGAAGAAGGAGAAAAAATGGTTTTGGAGAAT
AACTTCTTCGTGGAAACCATGTTGCCATCAAAAATCATGAGAAAGTTAGAACCAGAAGAATTTGCAGCATATCTTGAACCATTCAAAGAGAAAGGTGAAGTTCGTCGTCCAACATTATCATGGCCTCGT
GAAATCCCGTTAGTAAAAGGTGGTAAACCTGACGTTGTACAAATTGTTAGGAATTATAATGCTTATCTACGTGCAAGTGATGATTTACCAAAAATGTTTATTGAATCGGACCCAGGATTCTTTTCCAAT
GCTATTGTTGAAGGTGCCAAGAAGTTTCCTAATACTGAATTTGTCAAAGTAAAAGGTCTTCATTTTTCGCAAGAAGATGCACCTGATGAAATGGGAAAATATATCAAATCGTTCGTTGAGCGAGTTCTC
AAAAATGAACAATAATTCTAGAGCGGCCGCTTCGAGCAGACATGATAAGATACATTGATGAGTTTGGACAAACCACAACTAGAATGCAGTGAAAAAAATGCTTTATTTGTGAAATTTGTGATGCTATTG
CTTTATTTGTAACCATTATAAGCTGCAATAAACAAGTTAACAACAACAATTGCATTCATTTTATGTTTCAGGTTCAGGGGGAGGTGTGGGAGGTTTTTTAAAGCAAGTAAAACCTCTACAAATGTGGTA
AAATCGATAAGGATCCAGGTGGCACTTTTCGGGGAAATGTGCGCGGAACCCCTATTTGTTTATTTTTCTAAATACATTCAAATATGTATCCGCTCATGAGACAATAACCCTGATAAATGCTTCAATAAT
ATTGAAAAAGGAAGAGTATGAGTATTCAACATTTCCGTGTCGCCCTTATTCCCTTTTTTGCGGCATTTTGCCTTCCTGTTTTTGCTCACCCAGAAACGCTGGTGAAAGTAAAAGATGCTGAAGATCAGT
TGGGTGCACGAGTGGGTTACATCGAACTGGATCTCAACAGCGGTAAGATCCTTGAGAGTTTTCGCCCCGAAGAACGTTTTCCAATGATGAGCACTTTTAAAGTTCTGCTATGTGGCGCGGTATTATCCC
GTATTGACGCCGGGCAAGAGCAACTCGGTCGCCGCATACACTATTCTCAGAATGACTTGGTTGAGTACTCACCAGTCACAGAAAAGCATCTTACGGATGGCATGACAGTAAGAGAATTATGCAGTGCTG
CCATAACCATGAGTGATAACACTGCGGCCAACTTACTTCTGACAACGATCGGAGGACCGAAGGAGCTAACCGCTTTTTTGCACAACATGGGGGATCATGTAACTCGCCTTGATCGTTGGGAACCGGAGC
TGAATGAAGCCATACCAAACGACGAGCGTGACACCACGATGCCTGTAGCAATGGCAACAACGTTGCGCAAACTATTAACTGGCGAACTACTTACTCTAGCTTCCCGGCAACAATTAATAGACTGGATGG
AGGCGGATAAAGTTGCAGGACCACTTCTGCGCTCGGCCCTTCCGGCTGGCTGGTTTATTGCTGATAAATCTGGAGCCGGTGAGCGTGGGTCTCGCGGTATCATTGCAGCACTGGGGCCAGATGGTAAGC
CCTCCCGTATCGTAGTTATCTACACGACGGGGAGTCAGGCAACTATGGATGAACGAAATAGACAGATCGCTGAGATAGGTGCCTCACTGATTAAGCATTGGTAACTGTCAGACCAAGTTTACTCATATA
TACTTTAGATTGATTTAAAACTTCATTTTTAATTTAAAAGGATCTAGGTGAAGATCCTTTTTGATAATCTCATGACCAAAATCCCTTAACGTGAGTTTTCGTTCCACTGAGCGTCAGACCCCGTAGAAA
AGATCAAAGGATCTTCTTGAGATCCTTTTTTTCTGCGCGTAATCTGCTGCTTGCAAACAAAAAAACCACCGCTACCAGCGGTGGTTTGTTTGCCGGATCAAGAGCTACCAACTCTTTTTCCGAAGGTAA
CTGGCTTCAGCAGAGCGCAGATACCAAATACTGTTCTTCTAGTGTAGCCGTAGTTAGGCCACCACTTCAAGAACTCTGTAGCACCGCCTACATACCTCGCTCTGCTAATCCTGTTACCAGTGGCTGCTG
CCAGTGGCGATAAGTCGTGTCTTACCGGGTTGGACTCAAGACGATAGTTACCGGATAAGGCGCAGCGGTCGGGCTGAACGGGGGGTTCGTGCACACAGCCCAGCTTGGAGCGAACGACCTACACCGAAC
TGAGATACCTACAGCGTGAGCTATGAGAAAGCGCCACGCTTCCCGAAGGGAGAAAGGCGGACAGGTATCCGGTAAGCGGCAGGGTCGGAACAGGAGAGCGCACGAGGGAGCTTCCAGGGGGAAACGCCT
GGTATCTTTATAGTCCTGTCGGGTTTCGCCACCTCTGACTTGAGCGTCGATTTTTGTGATGCTCGTCAGGGGGGCGGAGCCTATGGAAAAACGCCAGCAACGCGGCCTTTTTACGGTTCCTGGCCTTTT
GCTGGCCTTTTGCTCACATGGCTCGAC

Table S4: Distribution of predicted RNA duplex motifs GC-helix, AU-helix, GC-duplex, and AU-duplex. RNA sequences were classified in five groups a) human Ago-like Argonautes, b) none-human Argonautes (Ago- and PIWI-like), c) human mRNAs, d) non-coding RNAs, and e) shuffled mRNAs (for accession numbers of RNA sequences see Table S2). Each sequence (except for non-coding RNAs) is sub-divided into three regions (i) 5`-region (5`-UTR + 200 nt), (ii) rCDS (remaining coding sequence), and (iii) 3`-UTR (see also Figure 2B). Analysis of secondary structure motif abundance is based on three secondary structure prediction approaches (i) sliding window (window size = 800 nt, step width = 20 nt, based on Mfold 2.3), (ii) Mfold 3.4, and (iii) Sfold 2.2. Abundance of structural motifs is given in motifs per 100 nt. Numbers in brackets indicate absolute number of helices found in each group. Absolute numbers are not directly comparable among the three approaches, since not all sequences could be fold by Sfold 2.2 and Mfold 3.4, respectively, due to length restrictions for input sequences on the online servers . Parameters for definition of the four structural motifs are provided in Table S1.

**GC helix**[1]		**5`-region**			**rCDS**				**3`-UTR**	
	**sliding win (mfold2.3)**	**mfold3.4**	**sfold2.2**	**sliding win (mfold2.3)**	**mfold3.4**	**sfold2.2**	**sliding win (mfold2.3)**	**mfold3.4**	**sfold2.2**
hAgo	0.24 (7)	0.07 (2)	0.03 (1)	0 (0)	0.01 (1)	0.01 (1)	0 (0)	0 (0)	0 (0)
none-h Argo	0.07 (12)	0.06 (11)	0.04 (7)	0.00 (2)	0 (0)	0 (0)	0 (0)	0 (0)	0 (0)
human mRNA	0.08 (32)	0.06 (23)	0.04 (15)	0.01 (11)	0.00 (6)	0.00 (5)	0.00 (2)	0.00 (1)	0.00 (1)
shuffled mRNA	0.01 (4)	0.01 (4)	n/a	0.00 (1)	0.00 (1)	n/a	0.00 (4)	0.00 (1)	n/a
					**complete sequence**				
non-coding				0 (0)	0 (0)	0 (0)			

[1]≥ 8 uninterrupted GC pairs, strand distance ≥ 40 nt, ≥ 16 nt in linker region involved in base pairings

**AU helix**[2]		**5`-region**		**rCDS**				**3`-UTR**	
	**sliding win (mfold2.3)**	**mfold3.4**	**sfold2.2**	**sliding win (mfold2.3)**	**mfold3.4**	**sfold2.2**	**sliding win (mfold2.3)**	**mfold3.4**	**sfold2.2**
hAgo	0 (0)	0 (0)	0 (0)	0 (0)	0 (0)	0 (0)	0.03 (3)	0.02 (2)	0.03 (2)
none-h Argo	0.01 (1)	0.01 (1)	0.01 (1)	0.00 (3)	0.00 (5)	0.00(4)	0.02 (10)	0.00 (2)	0.00(6)
human mRNA	0.00 (1)	0.02 (7)	0.01 (5)	0 (0)	0.00 (7)	0 (0)	0.03 (35)	0.03 (33)	0.02 (17)
shuffled mRNA	0 (0)	0.01 (4)	n/a	0.00 (1)	0 (0)	n/a	0 (0)	0 (0)	n/a
					**complete sequence**				
non-coding				0.02 (70)	0.03 (15)	0.02 (2)			

[2]≥ 8 uninterrupted AU pairs, strand distance ≥ 40 nt, ≥ 16 nt in linker region involved in base pairings

GC duplex[4]		5`-region		rCDS			3`-UTR	
	sliding win (mfold2.3)	mfold3.4	sfold2.2	sliding win (mfold2.3)	mfold3.4	sfold2.2	sliding win (mfold2.3)	mfold3.4	sfold2.2
human mRNA, n=12	0.01 (4)	0.01 (2)	0.01 (2)	0 (0)	0 (0)	0 (0)	0 (0)	0 (0)	0 (0)
human mRNA, n=11	0.02 (9)	0.01 (4)	0.01 (4)	0 (0)	0 (0)	0 (0)	0 (0)	0 (0)	0 (0)
human mRNA, n=10	0.02 (9)	0.01 (4)	0.01 (4)	0 (0)	0 (0)	0 (0)	0 (0)	0 (0)	0 (0)
human mRNA, n=9	0.05 (18)	0.03 (10)	0.02 (8)	0.00 (1)	0.00 (1)	0.00 (1)	0 (0)	0 (0)	0 (0)
human mRNA, n=8	0.1 (37)	0.07 (28)	0.06 (20)	0.01 (13)	0.00 (7)	0.00 (6)	0.00 (2)	0.00 (1)	0.00 (1)
human mRNA, n=7	0.2 (75)	0.18 (67)	0.12 (42)	0.02 (37)	0.01 (19)	0.01 (13)	0.00 (4)	0.00 (3)	0.00 (3)
human mRNA, n=6	0.48 (181)	0.42 (159)	0.25 (91)	0.08 (146)	0.04 (70)	0.03 (51)	0.02 (22)	0.01 (9)	0.01 (9)
human mRNA, n=5	1.16 (438)	1.04 (391)	0.55 (197)	0.34 (591)	0.13 (231)	0.10 (167)	0.13 (125)	0.05 (51)	0.04 (34)
shuffled mRNA, n=9	0.00 (2)	0.00 (1)	n/a	0 (0)	0 (0)	n/a	0 (0)	0 (0)	n/a
shuffled mRNA, n=8	0.01 (4)	0.01 (4)	n/a	0.00 (1)	0.00 (1)	n/a	0.00 (4)	0.00 (1)	n/a
shuffled mRNA, n=7	0.02 (13)	0.03 (20)	n/a	0.01 (21)	0.01 (13)	n/a	0.02 (23)	0.01 (10)	n/a
shuffled mRNA, n=6	0.08 (60)	0.09 (69)	n/a	0.08 (122)	0.04 (66)	n/a	0.06 (81)	0.03 (40)	n/a
shuffled mRNA, n=5	0.36 (288)	0.40 (321)	n/a	0.37 (604)	0.18 (289)	n/a	0.31 (403)	0.12 (151)	n/a

GC duplex[5]	complete sequence		
	sliding win (mfold2.3)	mfold3.4	sfold2.2
non-coding, n= 8	0 (0)	0 (0)	0 (0)
non-coding, n=7	0.00 (2)	0.00 (1)	0.00 (1)
non-coding, n=6	0.01 (23)	0.02 (14)	0.02 (7)
non-coding, n=5	0.05 (160)	0.12 (79)	0.08 (24)

[4]≥ n uninterrupted GC pairs

AU duplex[5]		5`-region			rCDS			3`-UTR	
	sliding win (mfold2.3)	mfold3.4	sfold2.2	sliding win (mfold2.3)	mfold3.4	sfold2.2	sliding win (mfold2.3)	mfold3.4	sfold2.2
human mRNA, n=12	0 (0)	0.01 (3)	0.01 (4)	0 (0)	0.00 (4)	0 (0)	0.01 (10)	0.01 (9)	0.00 (4)
human mRNA, n=11	0 (0)	0.01 (3)	0.01 (4)	0 (0)	0.00 (4)	0 (0)	0.01 (15)	0.01 (13)	0.01 (7)
human mRNA, n=10	0 (0)	0.01 (3)	0.01 (4)	0 (0)	0.00 (4)	0 (0)	0.02 (20)	0.02 (18)	0.01 (11)
human mRNA, n=9	0.00 (1)	0.01 (3)	0.01 (4)	0 (0)	0.00 (4)	0 (0)	0.03 (35)	0.03 (29)	0.02 (17)
human mRNA, n=8	0.01 (2)	0.02 (7)	0.01 (5)	0 (0)	0.00 (7)	0 (0)	0.06 (71)	0.05 (50)	0.03 (28)
human mRNA, n=7	0.01 (3)	0.02 (9)	0.01 (5)	0.01 (10)	0.01 (19)	0.00 (4)	0.13 (157)	0.08 (92)	0.05 (48)
human mRNA, n=6	0.03 (12)	0.05 (20)	0.03 (10)	0.03 (44)	0.03 (50)	0.01 (18)	0.31 (378)	0.16 (180)	0.10 (98)
human mRNA, n=5	0.10 (38)	0.18 (68)	0.06 (23)	0.10 (174)	0.11 (189)	0.04 (63)	0.71 (860)	0.33 (370)	0.19 (182)
shuffled mRNA, n=9	0 (0)	0 (0)	n/a	0 (0)	0 (0)	n/a	0 (0)	0 (0)	n/a
shuffled mRNA, n=8	0 (0)	0.01 (4)	n/a	0.00 (2)	0.00 (1)	n/a	0 (0)	0 (0)	n/a
shuffled mRNA, n=7	0.00 (3)	0.01 (10)	n/a	0.01 (10)	0.00 (2)	n/a	0 (1)	0 (0)	n/a
shuffled mRNA, n=6	0.02 (14)	0.03 (21)	n/a	0.03 (41)	0.01 (15)	n/a	0.01 (13)	0.01 (9)	n/a
shuffled mRNA, n=5	0.11 (84)	0.10 (82)	n/a	0.12 (201)	0.04 (69)	n/a	0.09 (117)	0.04 (48)	n/a

AU duplex[5]		complete sequence	
	sliding win (mfold2.3)	mfold3.4	sfold2.2
non-coding, n= 12	0.00 (4)	0.00 (2)	0.00 (1)
non-coding, n= 9	0.01 (34)	0.01 (6)	0.01 (2)
non-coding, n= 8	0.03 (104)	0.03 (20)	0.01 (3)
non-coding, n=7	0.09 (281)	0.07 (43)	0.01 (4)
non-coding, n=6	0.22 (281)	0.13 (87)	0.04 (11)
non-coding, n=5	0.60 (1955)	0.41 (270)	0.10 (32)

[5]≥ n uninterrupted AU pairs
